# Sphingomyelin Synthase 2 Promotes Endothelial Dysfunction by Inducing Endoplasmic Reticulum Stress

**DOI:** 10.3390/ijms20122861

**Published:** 2019-06-12

**Authors:** Lingyue Hua, Na Wu, Ruilin Zhao, Xuanhong He, Qian Liu, Xiatian Li, Zhiqiang He, Lehan Yu, Nianlong Yan

**Affiliations:** 1 Department of Biochemistry and Molecular Biology, School of Basic Medical Science, Nanchang University, Nanchang 330006, Jiangxi, China; hly3288551238@163.com (L.H.); wn13907096825@163.com (N.W.); zrl953226930@163.com (R.Z.); hxhxhong@163.com (X.H.); liucandice0412@163.com (Q.L.); 15807939939@163.com (X.L.); hzq3231103954@163.com (Z.H.); 2 School of Basic Medical Experiments Center, Nanchang University, Nanchang 330006, Jiangxi, China; yulehan@sohu.com

**Keywords:** atherosclerosis, sphingomyelin synthase 2, endothelial dysfunction, endoplasmic reticulum stress, β-catenin

## Abstract

Endothelial dysfunction (ED) is an important contributor to atherosclerotic cardiovascular disease. Our previous study demonstrated that sphingomyelin synthase 2 (SMS2) promotes ED. Moreover, endoplasmic reticulum (ER) stress can lead to ED. However, whether there is a correlation between SMS2 and ER stress is unclear. To examine their correlation and determine the detailed mechanism of this process, we constructed a human umbilical vein endothelial cell (HUVEC) model with SMS2 overexpression. These cells were treated with 4-PBA or simvastatin and with LiCl and salinomycin alone. The results showed that SMS2 can promote the phosphorylation of lipoprotein receptor-related protein 6 (LRP6) and activate the Wnt/β-catenin pathway and that activation or inhibition of the Wnt/β-catenin pathway can induce or block ER stress, respectively. However, inhibition of ER stress by 4-PBA can decrease ER stress and ED. Furthermore, when the biosynthesis of cholesterol is inhibited by simvastatin, the reduction in intracellular cholesterol coincides with a decrease in ER stress and ED. Collectively, our results demonstrate that SMS2 can activate the Wnt/β-catenin pathway and promote intracellular cholesterol accumulation, both of which can contribute to the induction of ER stress and finally lead to ED.

## 1. Introduction

Angiocardiopathy is a significant cause of death in many countries. Atherosclerosis (AS), which is a major cause of angiocardiopathy, is an inflammatory disease that leads to clogged arteries [1]. Additionally, endothelial dysfunction (ED) plays a crucial role in the pathogenesis of atherosclerotic cardiovascular disease [2]. Various harmful stimuli, such as oxidative stress and inflammation, can lead to ED, and reactive oxygen species (ROS) can induce oxidative stress, which plays an essential role in ED [3,4]. Since H_2_O_2_ is a key ROS, in this research, human umbilical vein endothelial cells (HUVECs) were treated with H_2_O_2_ to establish a cell model of oxidative stress [5].

Sphingomyelin (SM) is a type of sphingolipid that is important for the composition of biological membranes and plasma lipoproteins [6,7]. The production of SM requires many enzymatic reactions, and sphingomyelin synthase (SMS), which has two isoforms (sphingomyelin synthase 1 (SMS1) and sphingomyelin synthase 2 (SMS2)), is a critical enzyme in the final step of the production of SMS [8]. Studies have shown that SM participates in AS [9,10,11]. The level of SM in normal arterial tissue is significantly lower than that in atherosclerotic lesions [10]. Chemical inhibition of sphingolipid biosynthesis can markedly reduce the size of AS lesions in ApoE KO (apolipoprotein E knock out) mice [11]. These studies have mainly concentrated on the impact of SMS on reverse cholesterol transport and foam cell production in the process of AS development. However, our recent study indicated that SMS2 can also promote ED by activating the Wnt/β-catenin pathway under conditions of oxidative stress [12]. The typical Wnt/β-catenin pathway plays a critical role in many physiological processes, such as tissue patterning, the specification of cell fate, and cell proliferation [13,14]. During the process of transmembrane signal transduction, Wnt combines with the transmembrane receptor frizzled (FZD) and the coreceptor low-density lipoprotein receptor-related protein 6 (LRP6) to induce the phosphorylation of LRP6, which is necessary for activating the downstream Wnt/β-catenin pathway [13,14]. Since ED plays a crucial role in the initiation of AS [2], the Wnt/β-catenin pathway also participates in AS and its development [15,16,17,18,19,20]. For example, Bhatt et al. found that Wnt5a expression in serum from atherosclerotic patients is associated with the severity of atherosclerotic lesions [17,18]. However, the detailed mechanism of SMS2 related with the Wnt/β-catenin pathway and ED (AS) is not clear. 

The endoplasmic reticulum (ER) is an organelle that participates in protein folding, calcium homeostasis, and lipid biosynthesis. Many factors, including hyperlipidemia and oxidative stress, can disrupt homeostasis in the ER and the unfolded protein response (UPR) to induce ER stress [21,22]. During the process of ER stress, the chaperone GRP78 dissociates from PERK, IRE1, and ATF6, activating their downstream signaling pathways and influencing homeostasis in cells [23,24]. ER stress is strongly linked to the development of AS, and expression of GRP78, p-PERK, p-IRE1, ATF6, and CHOP is increased in ApoE knockout mice [25,26]. In addition, many atherogenic risk factors can activate ER stress during the initial stages of AS, strengthening ED and AS [27,28]. Undoubtedly, ER stress is involved in not only AS but also ED.

Importantly, SMS2, ER stress, and the Wnt/β-catenin pathway are all related to ED. Although our previous study revealed that SMS2 can lead to ED by inducing the Wnt/β-catenin pathway, the relationship between SMS2 and ER stress and the specific mechanism by which SMS2 regulates the Wnt/β-catenin pathway needs further research. Therefore, we aimed to identify the mechanism using HUVECs.

## 2. Results

### 2.1. SMS2 Can Activate ER Stress

**Figure 1:**
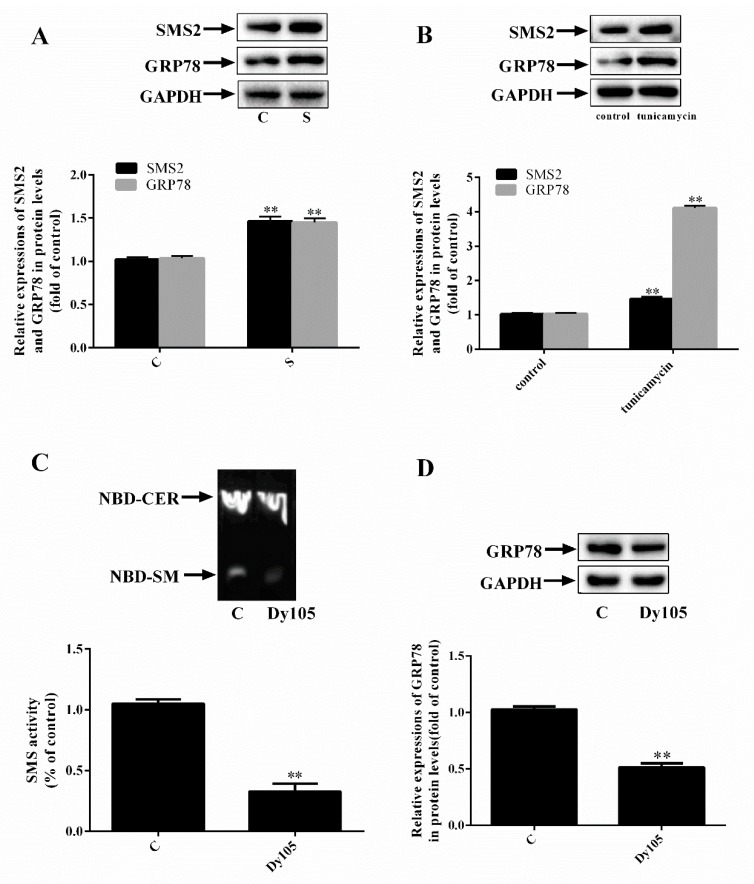
Sphingomyelin synthase 2 (SMS2) overexpression activates endoplasmic reticulum (ER) stress. Either a SMS2 overexpressed plasmid was used to transfect human umbilical vein endothelial cells (HUVECs) or the cells were treated with tunicamycin (10 µg/mL). (**A**) The protein levels of SMS2 and GRP78 were measured by a western blot analysis. (**B**) The protein levels of SMS2 and GRP78 were measured by a western blot analysis. (**C**) SMS activity was measured by thin-layer chromatography. (**D**) The expression of GRP78 was measured by a western blot analysis. *n* = 3, * *p* < 0.05, and ** *p* < 0.001 vs. the C group. (**A**) C, transfected with empty plasmids; S, cells overexpressing SMS2. (**C**,**D**) C, control group; Dy105, cells treated with Dy105. (**C**) NBD-CER, Norbornadiene -ceramide, NBD-SM, Norbornadiene-sphingomyelin.

Both ER stress and SMS2 are associated with ED; however, the mechanism involved needs further study. First, we established SMS2 overexpression in HUVECs. These results (Figure 1A) showed that the amounts of SMS2 and the ER stress marker protein GRP78 in the S group were upregulated compared with those in the C group (C, transfected with empty plasmids; S, cells overexpressing SMS2; *p* < 0.001; *n* = 3). Furthermore, an ER stress cell model was established by treating cells with tunicamycin (10 µg/mL) for 24 h. The results (Figure 1B) verified that expression of SMS2 and GRP78 was upregulated by 46.3% and 44.8% in the tunicamycin group compared with that in the C group, respectively (*p* < 0.001; *n* = 3). To rule out the possibility that endoplasmic reticulum stress was not caused by protein overload but the overexpression of SMS2, we treated the HUVECs with 20 µmol/L Dy105 (an inhibitor of SMS2). We then measured the activity of SMS2 and expression of GRP78. Based on the data presented in Figure 1C, we identified that the SMS enzyme activity was markedly decreased compared with that in the C group; this activity was decreased by 60.09% compared with that in the C group (*p* <0.001; *n* = 3). In addition, the expression of GRP78 was decreased by 40.5% (Figure 1D; *p* < 0.001; *n* = 3). These findings demonstrate that ER stress is significantly induced by SMS2.

### 2.2. SMS2 Can Trigger ER Stress by Provoking the Wnt/β-Catenin Pathway

**Figure 2:**
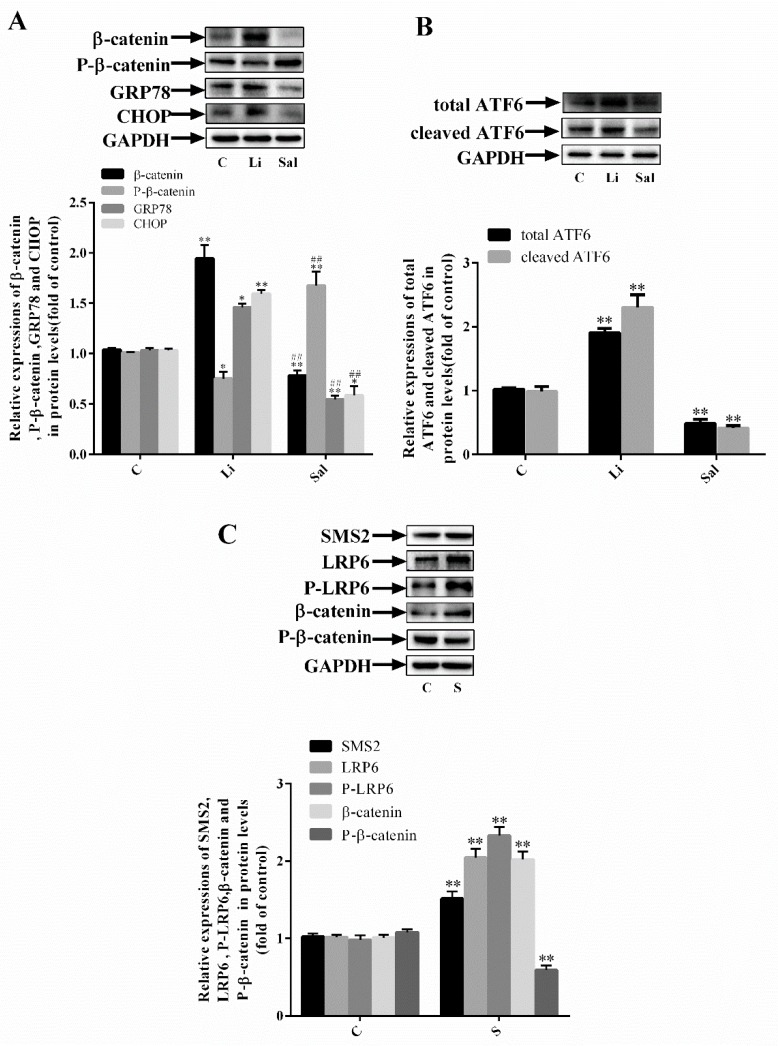
SMS2 can trigger ER stress by inducing the Wnt/β-catenin pathway. (**A**) Western blot analysis detected the protein expression of β-catenin, phosphorylated β-catenin, GRP78, and CHOP. (**B**) Western blotting analysis detected the protein expression of the total ATF6 and cleaved ATF6. (**C**) Western blotting analysis detected the protein expression of SMS2, β-catenin, phosphorylated β-catenin, lipoprotein receptor-related protein 6 (LRP6), and phosphorylated LRP6. *n* = 3, * *p* < 0.05 and ** *p* < 0.001 vs. the C group; ^##^ *p* < 0.001 vs. the Li group. C, control cells; S, cells overexpressing SMS2; Li, LiCl group, control cells treated with LiCl (40 µmol/L) for 24 h; Sal, salinomycin group, control cells treated with salinomycin (5 µmol/L) for 24 h.

To further explore the specific mechanism of SMS2-induced ER stress, LiCl (40 µmol/L) and salinomycin (5 µmol/L) were used to activate and inhibit the Wnt/β-catenin pathway, respectively. The results showed that, compared with the C group, the levels of the ER stress-related proteins GRP78, CHOP, and β-catenin were upregulated by 45.94%, 59.51%, and 94.55% in the Li group and decreased by 45.5%, 41.36%, and 28.4% in the Sal group, respectively (Figure 2A: C, control cells; Sal, salinomycin; Li, LiCl group; *p* < 0.001; *n* = 3). However, relative expression of phosphorylated β-catenin was decreased by 24.9% in the Li group compared with that in the C group and increased by 67.7% in the Sal group compared with that in the C group (Figure 2A: *p* < 0.05; *n* = 3). Additionally, we found that the expression of the total ATF6 and cleaved ATF6 (P50) were significantly increased by 96.5% and 126.3% compared with the C group, by activating the Wnt/β-catenin pathway. On the contrary, in the Sal group the expression of the total ATF6 and cleaved ATF6 were significantly decreased by 50.6% and 60.2% compared with the C group. (Figure 2B: *p* < 0.05; *n* = 3). These results suggest that the provocation of Wnt/β-catenin can induce ER stress and that the suppression of Wnt/β-catenin can inhibit ER stress. Previous papers published by the authors have shown that SMS2 can cause dysfunction in endothelial cells by inducing the Wnt/β-catenin pathway. As shown in Figure 2C, compared with the C group, relative expression of β-catenin, phosphorylated LRP6, and LRP6 was upregulated by 101.9%, 132.9%, and 104.6% in the SMS2 group, respectively (*p* < 0.001; *n* = 3). In contrast, relative expression of phosphorylated β-catenin was reduced by 45.7%. These results suggest that SMS2 is able to trigger ER stress by inducing Wnt/β-catenin signaling.

### 2.3. Inhibition of ER Stress Can Decrease SMS2-Induced ED

**Figure 3:**
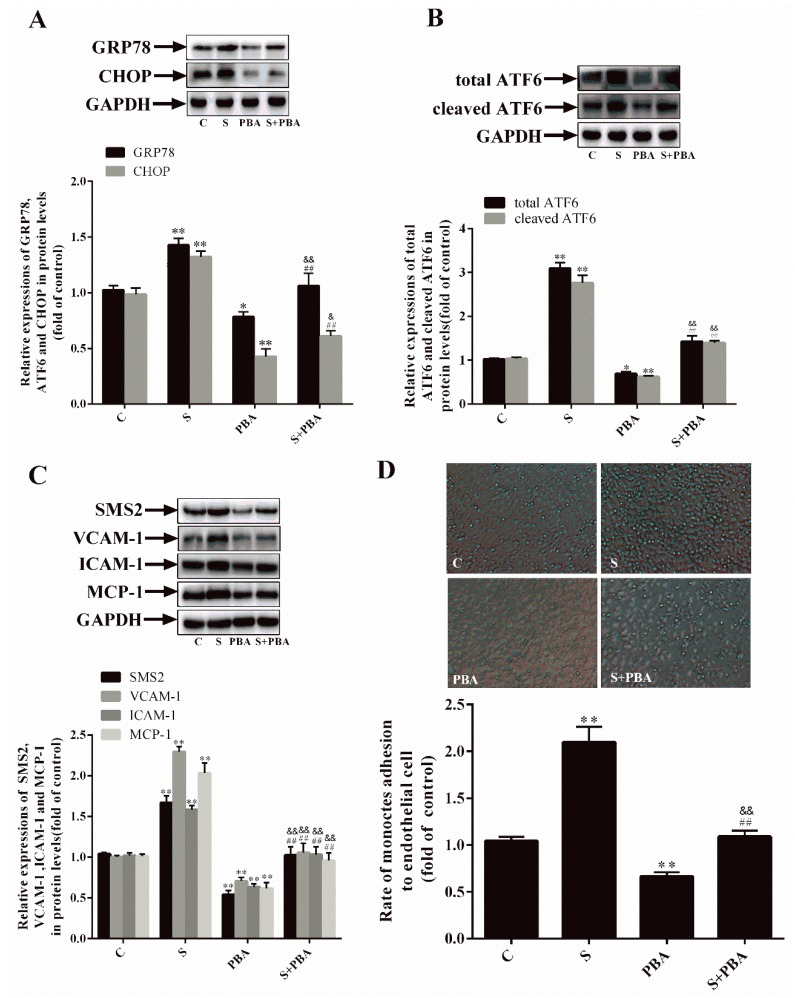
SMS2 can induce ER stress and endothelial dysfunction (ED) by inducing the Wnt/β-catenin pathway. (**A**) The protein levels of GRP78 and CHOP were determined by a western blot analysis. (**B**) The protein levels of the total ATF6 and cleaved ATF6 were determined by a western blot analysis. (**C**) The protein levels of VCAM-1, ICAM-1, and MCP-1 were determined by a western blot analysis. (**D**) The adhesion ratio of THP-1 cells to HUVECs (magnification 40×). *n* = 3, * *p* < 0.05, and ** *p* < 0.001 vs. the C group; ^##^ *p* < 0.001 vs. the S group. ^&^ *p* < 0.05 and ^&&^ *p* < 0.001 vs. the PBA group. C, cells transfected with empty plasmids treated with H_2_O_2_ (450 µmol/L) for 24 h; S, cells overexpressing SMS2 treated with H_2_O_2_ (450 µmol/L) for 24 h; PBA, empty plasmids treated with 4-PBA (10 mmol/L) for 24 h and then treated with H_2_O_2_ (450 µmol/L) for 24 h; S+PBA, cells overexpressing SMS2 treated with 4-PBA (10 mmol/L) for 24 h and then treated with H_2_O_2_ (450 µmol/L) for 24 h.

To prove the correlation between SMS2 and ER stress, cells were transfected with an empty plasmid or an SMS2 overexpression plasmid, treated with the ER stress inhibitor 4-PBA for 24 h, and treated with H_2_O_2_ for 24 h to establish an oxidative stress model. The results indicated that the GRP78 and CHOP protein expression levels in the S group were increased by 42.8% and 32.3%, respectively, compared with those in the C group. In the PBA group, the GRP78 and CHOP protein expression levels were significantly decreased (by 21.6% and 57.4%, respectively) compared with those in the C group. Furthermore, the total ATF6 and cleaved ATF6 protein expression levels in the S group were upregulated by 210.7% and 163.3% and downregulated by 32.1% and 40.2% in the PBA group, respectively, compared with those in the C group. In particular, in the S+PBA group, the levels of GRP78, CHOP, total ATF6, and cleaved ATF6 were markedly increased compared with those in the PBA group and down-regulated compared with those in the S group (Figure 3A,B: C, cells transfected with empty plasmids; S, cells overexpressing SMS2; PBA, empty plasmids treated with 4-PBA (10 mmol/L) for 24 h; S+PBA, cells overexpressing SMS2 treated with 4-PBA (10 mmol/L) for 24 h; and all cells were treated with H_2_O_2_ (450 µmol/L) for 24 h. *p* < 0.001, *n* = 3). These data suggest that SMS2 can induce but that PBA inhibits ER stress.

We then investigated the relationship among SMS2, ER stress, and ED. The results (Figure 3C) suggest that, compared with the transfection with the empty plasmid in the C group, the transfection with the SMS2 overexpression plasmid activated ER stress and increased the expression of the adhesion-related molecules ICAM-1, VCAM-1, and MCP-1 by 58.1%, 12.6%, and 103.2%, respectively. In contrast, the levels of these adhesion-related molecules were decreased by 42.8%, 29.3%, and 36.6% after the inhibition of ER stress by 4-PBA, compared with those in the C group without treatment. In addition, in the S+PBA group, the levels of ICAM-1, VCAM-1, and MCP-1 were markedly increased compared with those in the PBA group and down-regulated compared with those in the S group (*p* < 0.001, *n* = 3). Monocyte adhesion reflects the degree of cell damage that can lead to ED. As illustrated in Figure 3D, the adhesion ability in the S group was observably increased (by 100.96%) compared with that in the C group (Figure 3D: *p* < 0.05; *n* = 3), though the adhesion ability in the PBA group was significantly reduced (by 33.66%) compared with that in the C group (Figure 3D: *p* < 0.05; *n* = 3). These results demonstrate that the repression of ER stress can repress ED and that SMS2 can induce ED via ER stress.

### 2.4. Simvastatin Can Attenuate the ER Stress Induced by SMS2

**Figure 4:**
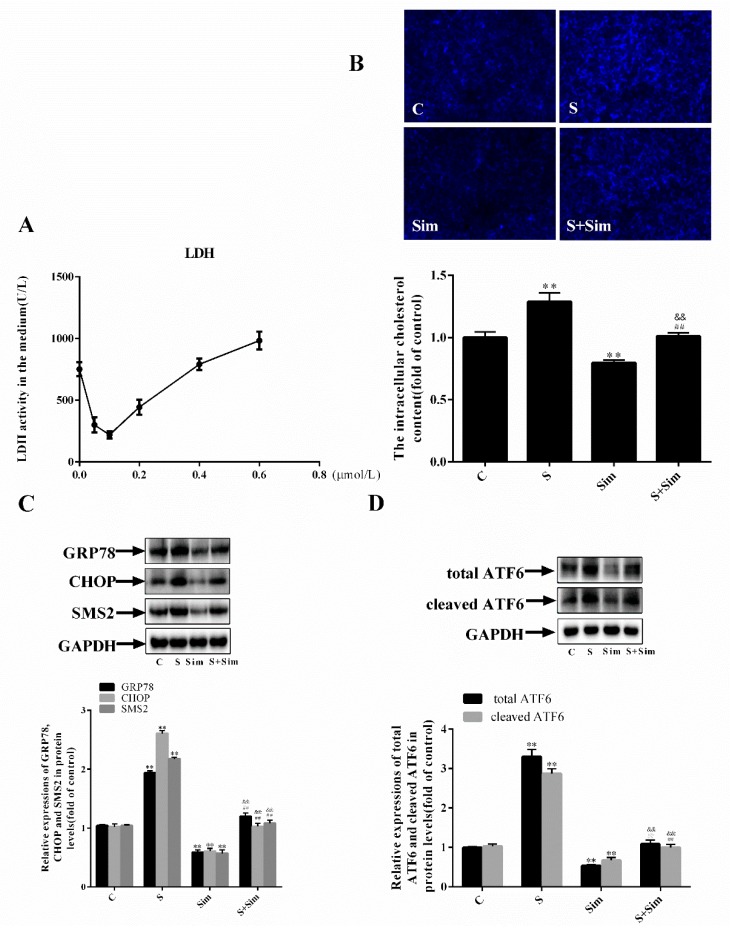
Overexpression of SMS2 can lead to ER stress by increasing the deposition of intracellular cholesterol. (**A**) HUVECs were treated with simvastatin at different doses (0, 0.05, 0.1, 0.2, 0.4, and 0.6 µmol/L) for 24 h, and the level of LDH in the cellular medium was detected. (**B**) The accumulation of ER cholesterol after filipin staining was visualized under a fluorescence microscope (magnification 40×). (**C**) The protein levels of GRP78 and CHOP were determined by a western blot analysis. (**D**) The protein levels of the total ATF6 and cleaved ATF6 were determined by a western blot analysis. *n* = 3, * *p* < 0.05, and ** *p* < 0.001 vs. the C group; ^##^ *p* < 0.001 vs. the S group. ^&&^ *p* < 0.001 vs. the Sim group. C, cells transfected with empty plasmids treated with H_2_O_2_ (450 µmol/L) for 24 h; S, cells overexpressing SMS2 treated with H_2_O_2_ (450 µmol/L) for 24 h; Sim, empty plasmids treated with simvastatin (0.1 µmol/L) for 24 h and then treated with H_2_O_2_ (450 µmol/L) for 24 h; S+Sim, cells overexpressing SMS2 treated with simvastatin (0.1 µmol/L) for 24 h and then treated with H_2_O_2_ (450 µmol/L) for 24 h.

To determine whether the intracellular accumulation of cholesterol is affected by SMS2, the following experiments were performed. HUVECs were treated with different doses of simvastatin to reduce intracellular cholesterol synthesis. The results showed that the activity of LDH (lactic dehydrogenase) and a degree of cell injury were the lowest at the 0.1 µmol/L dose; therefore, the final dose of simvastatin used was 0.1 µmol/L (Figure 4A: *p* < 0.001, *n* = 3). Subsequently, the cells were stained with filipin. The results shown in Figure 4B reveal that the intracellular cholesterol accumulation in the S group was increased by 28.8% compared with that in the C group and decreased by 20.5% in the Sim group compared with that in the C group. In addition, the cholesterol accumulation in the S+Sim group was increased by 20.2% compared with that in the Sim group and decreased by 23.1% compared with that in the S group (C, cells transfected with empty plasmids; S, cells overexpressing SMS2; Sim, empty plasmids treated with simvastatin (0.1 µmol/L) for 24 h; S+Sim, cells overexpressing SMS2 treated with simvastatin (0.1 µmol/L) for 24 h; all the cells were treated with H_2_O_2_ (450 µmol/L) for 24 h, *p* < 0.001; *n* = 3). These findings suggest that overexpression of SMS2 may contribute to intracellular cholesterol accumulation. Furthermore, we detected the proteins related to ER stress, and the results (Figure 4C,D) showed that the protein expression of GRP78, CHOP, SMS2, total ATF6, and cleaved ATF6, in the S group was increased by 93.6%, 160.9%, 117.6%, 235.4%, and 180.5%, respectively, compared with that in the C group. Expression levels of the GRP78, CHOP, SMS2, total ATF6, and cleaved ATF6 proteins in the Sim group were inhibited compared with those in the C group, indicating that simvastatin can inhibit ER stress. In the S+Sim group, the levels of GRP78, CHOP, total ATF6, and cleaved ATF6 were markedly increased compared with those in the Sim group but reduced compared with those in the S group (*p* < 0.001, *n* = 3). These findings demonstrate that overexpression of SMS2 can cause cholesterol accumulation, which may contribute to ER stress.

### 2.5. Simvastatin Can Attenuate the Injury Induced by SMS2

**Figure 5:**
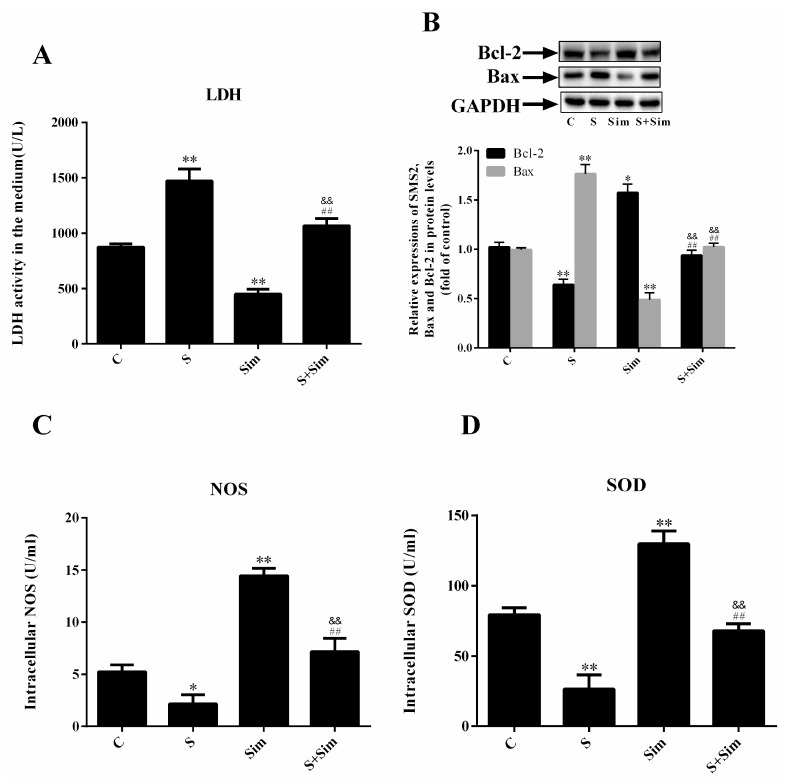
Overexpression of SMS2 can promote endothelial cell injury by increasing the deposition of intracellular cholesterol. (**A**) LDH, (**C**) NOS, and (**D**) SOD levels were measured with assay kits. (**B**) Western blot analysis detected the protein levels of SMS2, Bax, and Bcl-2. *n* = 3, * *p* < 0.05, and ** *p* < 0.001 vs. the C group; ^##^ *p* < 0.001 vs. the S group. ^&&^ *p* < 0.001 vs. the Sim group. C, cells transfected with empty plasmids treated with H_2_O_2_ (450 µmol/L) for 24 h; S, cells overexpressing SMS2 treated with H_2_O_2_ (450 µmol/L) for 24 h; Sim, empty plasmids treated with simvastatin (0.1 µmol/L) for 24 h and then treated with H_2_O_2_ (450 µmol/L) for 24 h; S+Sim, cells overexpressing SMS2 treated with simvastatin (0.1 µmol/L) for 24 h and then treated with H_2_O_2_ (450 µmol/L) for 24 h.

To further elucidate the effects of cholesterol accumulation on cell injury, we measured the LDH, SOD (superoxide dismutase), and NOS (nitric oxide synthase) content. The results showed that SOD and NOS production in the HUVECs in the S group was significantly reduced compared with that in the C group; however, treatment with simvastatin increased SOD and NOS production in the Sim group compared with that in the C group. Additionally, SOD and NOS production in the S+Sim group was upregulated compared with that in the S group but decreased compared with that in the Sim group (Figure 5C,D: *p* < 0.001, *n* = 3). Conversely, LDH activity showed the opposite trend (Figure 5A: *p* < 0.001, *n* = 3). These findings indicate that SMS2 overexpression can induce HUVEC injury due to intracellular cholesterol accumulation and that simvastatin has a protective effect on cells. Furthermore, compared with the C group, the results showed that, in the S group, the level of the pro-apoptotic gene Bax was increased by 76.5%, while the level of the anti-apoptotic gene Bcl-2 was reduced by 35.9%. This finding contrasts the results observed after the simvastatin treatment. In the Sim group, the level of the pro-apoptotic gene Bax was decreased by 51.3% compared with that in the C group, while the level of the anti-apoptotic gene Bcl-2 was increased by 57.2% compared with that in the C group (Figure 5B: *p* < 0.001, *n* = 3). These data indicate that overexpression of SMS2 can lead to ER stress and ED, due to cholesterol accumulation.

### 2.6. Simvastatin Can Attenuate the Adhesion Capacity Induced by SMS2

**Figure 6:**
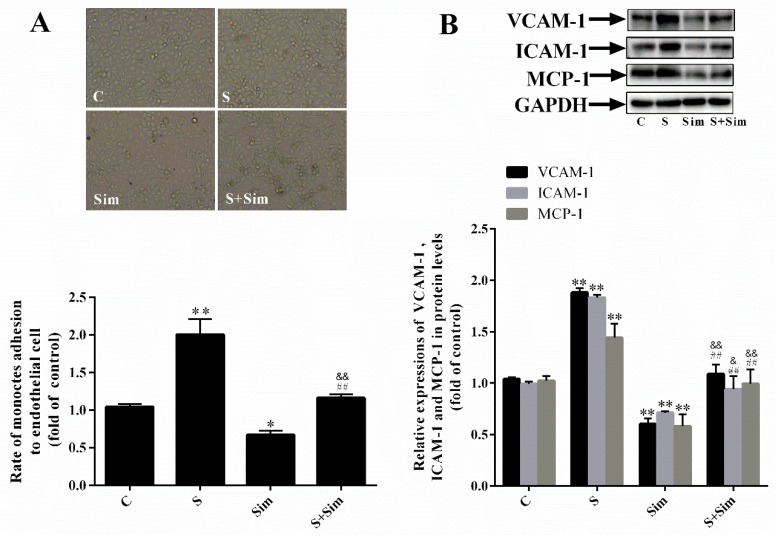
Overexpressed SMS2 increases the adhesion activity of HUVECs and THP-1 cells by increasing the deposition of intracellular cholesterol. (**A**) The adhesion ratio of THP-1 cells to HUVECs (magnification 40×). (**B**) Western blot analysis detected the protein level of VCAM-1, ICAM-1, and MCP-1. *n* = 3, * *p* < 0.05 and ** *p* < 0.001 vs. the C group; ^##^ *p* < 0.001 vs. the S group. ^&&^ *p* < 0.001 vs. the Sim group. C, cells treated with empty plasmids treated with H_2_O_2_ (450 µmol/L) for 24 h; S, cells overexpressing SMS2 treated with H_2_O_2_ (450 µmol/L) for 24 h; Sim, empty plasmids treated with simvastatin (0.1 µmol/L) for 24 h and then treated with H_2_O_2_ (450 µmol/L) for 24 h; S+Sim, cells overexpressing SMS2 treated with simvastatin (0.1 µmol/L) for 24 h and then treated with H_2_O_2_ (450 µmol/L) for 24 h.

We next analyzed the adhesion capacity of HUVECs and THP-1 cells to demonstrate the effects of simvastatin. Figure 6A shows that adhesion capacity in the S group was markedly increased (by 100.5%) compared with that in the C group (Figure 6A: *p* < 0.05; *n* = 3), adhesion capacity in the Sim group was evidently reduced (by 32.9%) compared with that in the C group (Figure 6A: *p* < 0.05; *n* = 3), and adhesion capacity in the S+Sim group was higher than that in the Sim group and lower than that in the S group. These findings suggest that simvastatin reduces cholesterol deposition, thus decreasing the cell adhesion capacity. Moreover, the results showed that, compared with transfection with the empty plasmid in the C group, transfection with the SMS2 overexpression plasmid increased cholesterol accumulation and expression of the adhesion molecules VCAM-1, ICAM-1, and MCP-1 by 88.1%, 83.2%, and 44.2%, respectively. Meanwhile, compared with the C group without treatment, the level of these adhesion-related molecules was decreased by 39.6%, 28.6%, and 41.8% after treatment with simvastatin (Figure 6B: *p* < 0.05; *n* = 3). Furthermore, S+Sim decreased expression of the adhesion-related molecules compared with simvastatin treatment in the S group (Figure 6B: *p* < 0.001; *n* = 3). These data suggest that simvastatin can attenuate the adhesion capacity induced by SMS2.

## 3. Discussion

Our previous study demonstrated that SMS2 can activate the Wnt/β-catenin pathway [12], but the detailed mechanism has remained unclear. Both SM and cholesterol are the main components of lipid rafts [29]. Therefore, changing the expression of SMS may affect the SM content in lipid rafts and, thus, influence transmembrane signal transduction. For example, Ding et al. found that overexpression of SMS can lead to the deposition of SM in cells and lipid rafts [30]. Lipid rafts play an essential role in physiological and biochemical processes as signaling “platforms”, such as the LPS receptor (lipopolysaccharides), TLR4, which must be recruited to lipid rafts to transduce extracellular signals to intracellular downstream signaling molecules [31]. During the process of Wnt/β-catenin signal transduction, the coreceptor LRP6 needs to be phosphorylated to disinhibit DKK1 (Dickkopf related protein 1) and form the FZD transmembrane protein receptor complex to promote signal transduction, and the binding between the LRP6 and FZD is affected by lipid rafts [32,33]. In this study, overexpressing SMS2 and both expressions of LRP6 and the phosphorylation of LRP6 were increased in HUVECs (Figure 2C). These results indicate that SMS2 may increase the phosphorylation of LRP6 in lipid rafts and decrease the degradation of LRP6 outside lipid rafts by promoting LRP6 endocytosis to lipid rafts [34,35]. Thereafter, the increase in expression and phosphorylation of LRP6 can lead to the provocation of the Wnt/β-catenin pathway, which contributes to ED (Figure 2 and Figure 6).

Previously, many studies have suggested that SMS2 is involved in AS by affecting reverse cholesterol transport [9,10,11]. However, we recently demonstrated that SMS2 also participates in ED by stimulating the Wnt/β-catenin signal pathway [19]. Meanwhile, some reports believe that ER stress can lead to ED [36,37], but the detailed correlation between these factors remains unexplored. Therefore, we transfected HUVECs with SMS2 overexpression plasmids. The results revealed that overexpression of SMS2 can increase expression of the ER stress marker protein, GRP78 (Figure 1). Interestingly, when ER stress was induced by tunicamycin, expression of SMS2 was also significantly increased (Figure 1). These results suggest that SMS2 can promote ER stress and that ER stress may regulate SMS2 expression.

**Figure 7:**
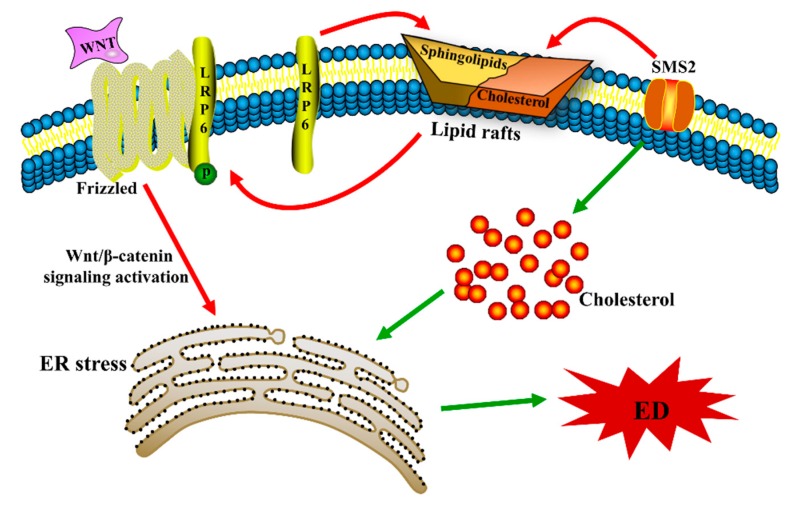
The possible mechanism by which endoplasmic reticulum stress is induced by SMS2. ER stress, endoplasmic reticulum stress; ED, endothelial dysfunction.

Since SMS2 can stimulate the Wnt/β-catenin pathway, we investigated whether SMS2 can induce ER stress, and subsequently ED, by activating the Wnt/β-catenin pathway in HUVECs. In this study, the Wnt/β-catenin pathway was activated or blocked by LiCl or salinomycin, respectively. The results revealed that activating Wnt/β-catenin signaling could decrease ER stress in the HUVECs and vice versa (Figure 2A,B). Mechanically, Zhang et al. suggested that the Wnt/β-catenin pathway blockage and β-catenin degradation result in the inhibition of the effect of LEF1 on ATF6 and activate ATF6-related ER stress [38]. These findings confirm that the activation of the Wnt/β-catenin pathway can promote ER stress. In fact, other studies have also shown that the Wnt/β-catenin pathway is negative with the ER stress in cancer cells, but our studies contradict these results [39,40]. These conflicting results suggest that the Wnt/β-catenin pathway has diverse functions in different types of cells. Previously, we proved that SMS2 can activate Wnt/β-catenin signaling; therefore, after the treatment with 4-PBA, compared with the simvastatin treatment in the S group, expression of ER stress-related proteins (GRP78, ATF6, and CHOP) and adhesion molecules (ICAM-1, VCAM-1, and MCP-1) and the adhesion activity were significantly decreased (Figure 3), suggesting that 4-PBA reverses the effects of SMS2 on ER stress and ED. These results further indicate that SMS2 can activate ER stress and promote oxidative stress-induced ED (Figure 3 and Figure 7).

The ER is not only involved in protein folding and modification but is also inextricably linked to the metabolism of lipids, such as cholesterol; therefore, lipid metabolism disorders can also trigger ER stress [41,42]. For example, recent animal and human studies have identified cholesterol deposition and ER stress activation as key players in the progression of many metabolic diseases [43]. Nonetheless, cholesterol deposition causes dysfunction in β-cells and promotes autophagy by activating ER stress [44,45]. In fact, SM and cholesterol can affect each other in cells and serum. For example, patients suffering from the Niemann–Pick disease (NPD-B) cannot synthesize SM due to defective SMase (sphingomyelinase), leading to the accumulation of SM and cholesterol in the liver [46]. Furthermore, we previously confirmed that overexpression of SMS in Huh7 cells markedly enhances the levels of intracellular sphingomyelin and cholesterol [47]. To investigate whether SMS2 expression can lead to the deposition of cholesterol in HUVECs, we measured intracellular cholesterol by filipin staining. The results suggested that in HUVECs, SMS2 overexpression can increase the intracellular cholesterol levels (Figure 4). The inhibition of cholesterol synthesis by simvastatin can reverse the deposition of cholesterol induced by SMS2, and expression of the ER stress-associated proteins, GRP78, CHOP, and ATF6, in the S+Sim group was also noticeably down-regulated compared with that in the S group (Figure 4). Finally, ED was found to be significantly attenuated (Figure 4 and Figure 5). SMS2 was shown to induce ER stress and ED by promoting intracellular cholesterol accumulation (Figure 6 and Figure 7). 

In conclusion, our results show that SMS2 (1) triggers the Wnt/β-catenin pathway and (2) promotes intracellular cholesterol accumulation, both of which contribute to the induction of ER stress and finally lead to ED. Although the mechanism of ED is very complex, we hope that our studies help to further elucidate the related mechanism.

## 4. Materials and Methods

### 4.1. Cell Culture and Reagents

HUVECs were obtained from the Cell Bank of Type Culture Collection of the Chinese Academy of Sciences (Shanghai, China) and cultured in Dulbecco’s modified Eagle’s medium (DMEM; cat. no. 12100-500; Beijing Solarbio Bioscience & Technology co., Ltd., Beijing, China) containing penicillin and streptomycin (100 U/mL and 0.1 mg/mL, respectively) and 10% certified fetal bovine serum (FBS; Biological Industries Israel Beit Haemek, KibbutzBeit Haemek, Israel) at 37 °C containing 5% CO2. In addition, the THP-1 cells (Cell Bank of Type Culture Collection of the Chinese Academy of Sciences, Shanghai, China) were grown in RPMI-1640 (cat. no. 31800; Beijing Solarbio Bioscience & Technology co., Ltd.) containing 10% FBS and incubated at 37 °C in a humidified atmosphere containing 5% CO2. Simvastin (cat. no. MB1222-S; Meilunbiotech Co., Ltd., Dalian, China), sodium 4-phenylbutyrate (4-PBA; cat. no. C1029659; Macklinbio co., Ltd., Shanghai, China), tunicamycin (cat. no. B7417; APExBIO co., Ltd., Shanghai, China), filipin (cat. no. B6034; APExBIO co., Ltd., Shanghai, China), and salinomycin (cat. no. HY-15597; Medchem Express co., Ltd., New Jersey, USA) were dissolved in DMSO (dimethyl sulfoxide; cat. no. 302A036; Beijing Solarbio Bioscience & Technology co., Ltd.). 

### 4.2. Transfection with an SMS2 Overexpression Plasmid

The SMS2-overexpression plasmid was provided by Dr. Tingbo Ding (School of Pharmacy, Fudan University, Shanghai, China) and used to transfect the HUVECs. In brief, the cells were seeded in culture flasks (Haote Technologies co., Ltd., Guangzhou, China), and at the time of transfection, the cells were grown to 90–95% confluency; the medium was replaced with the antibiotic-free DMEM medium (Biological Industries Israel Beit Haemek Ltd.). The SMS2 plasmid (4 µg; S group) or an empty control plasmid (4 µg; C group) was diluted with 400 µL DMEM (FBS-free and antibiotic-free medium), and 24 µL of Hieff Trans™ Liposomal Transfection Reagent was also diluted with 400 µL DMEM. After 5 min, the dilutions were gently mixed together and incubated at 37 °C for 20 min, and the mixture was added to each culture flask. Before adding the drugs, the medium was replaced with fresh DMEM (containing 10% FBS and antibiotics). The control and SMS2 groups were divided into two groups after 24 h, resulting in four small groups as follows: C (empty plasmid), S (SMS2), Sim (control + simvastatin), and S+Sim (SMS2 + simvastatin). Both the Sim and S+Sim groups were treated with simvastatin (0.1 µmol/L). The following set of groups were established: C (empty plasmid), S (SMS2), PBA (control + 4-PBA), and S+PBA (SMS2 + 4-PBA); both the PBA and S+PBA groups were treated with PBA (10 mmol/L). After 24 h, all groups were treated with H_2_O_2_ (500 µmol/L). Finally, after 24 h, the HUVECs were centrifuged at 1520× *g* for 5 min (at room temperature) as appropriate to collect all cells.

### 4.3. Measurement of the Degree of Oxidative Stress

HUVECs were cultured at a density of 1x10^5^/well in 6-well plates and incubated overnight at 37 °C. After the transfection, the cells were treated with simvastatin (0.1 µmol/L) for 24 h, and then the cells were treated with H_2_O_2_ (500 µmol/L) for 24 h. To collect the supernatant, the HUVECs were centrifuged (1520× *g*, 5 min, room temperature) and harvested, followed by digestion with trypsin, according to the protocol of the manufacturer. The levels of LDH (cat. no. A020-1; Nanjing Jiancheng Bioengineering Institute, Nanjing, China) and the levels of NOS (cat. no. A014-2-1; Nanjing Jiancheng Bioengineering Institute) and SOD (cat. no. A001-1-1; Nanjing Jiancheng Bioengineering Institute) activity were measured using a microplate reader (Thermo Fisher Scientific, Inc., Waltham, MA, USA). LDH was measured at a wavelength of 450 nm, and SOD and NOS were measured at a wavelength of 560 nm.

### 4.4. Filipin Staining

Cells were seeded in 24-well plates (Beaver Nano-Technologies co., Ltd., Suzhou, China) at a density of 2 × 10^4^ cells/well. Following the above transfection steps described in the HUVECs, the cells were treated with simvastatin (0.1 µmol/L) for 24 h. Subsequently, all cells were treated with H_2_O_2_ (500 µmol/L) for 24 h and washed with PBS (phosphate buffer saline) three times. Then, 10% paraformaldehyde was used to fix the cells at room temperature for 10 min; subsequently, the cells were washed with PBS three times again. To eliminate the paraformaldehyde, the cells were washed with PBS containing glycine (1.5 mg/mL), and then filipin was added in a dark room for 1 h. The cells were washed with PBS three times. Finally, we used UV excitation at 405 nm and confocal microscopy to observe the cholesterol aggregation.

### 4.5. Cell Adhesion Assay

Cells were seeded in 24-well plates in three replicates per group to obtain the average number of adhesive monocytes/well. Following the above transfection steps described in the HUVECs, THP-1 cells at a density of 5 × 10^3^/well were added and incubated for 2 h at 37 °C. The medium was discarded, and the non-adherent THP-1 cells were removed by washing with PBS three times. The adherent THP-1 cells were counted in a single field under a phase contrast inverted microscope (Magnification, 20×; Olympus IX71; Olympus corporation, Tokyo, Japan).

### 4.6. Western Blot Analysis

The total proteins were extracted from all groups by a radioimmunoprecipitation assay buffer (cat. no. ROO20; Beijing Solarbio Bioscience & Technology co., Ltd.), and the protein content was measured using a BCA assay kit (Bradford Protein Assay kit; cat. no. cW0014; Beijing Kangwei century Biotechnology co., Ltd., Beijing, China). Equal amounts of protein (~50 µg) were separated by 8–10% SDS-PAGE and transferred onto polyvinylidene fluoride membranes (Immobilon-P; EMd Millipore, Billerica, MA, USA). An equal transfer was examined by staining with Ponceau red (cat. no. CW0057S; Beijing Kangwei century Biotechnology co., Ltd.). The membranes were blocked with 5% skimmed milk or 5% BSA (bovine serum albumin) in TBS (phosphate buffer saline + Tween) for 1 h at room temperature and incubated with primary antibodies overnight at 4 °C in TBST containing 0.05% Tween 20 and 2% bovine serum albumin (cat. no. A8020; Beijing Solarbio Science & Technology co., Ltd.). Subsequently, the membranes were incubated with a 1:8,000-dilution of a horseradish peroxidase-conjugated secondary antibody for 1 h at room temperature. The peroxidase activity was visualized using an ECL kit (Bio-Rad Laboratories Inc., USA). GAPDH was used as a loading control. Anti-SMS2 (cat. no SA100531AA; 1:1,000) antibody was purchased from Abgent Biotech. (Suzhou co., Ltd., Suzhou, China). Anti-apoptosis-associated proteins B-cell lymphoma 2 (Bcl-2; cat. no. 60178-1-Ig; 1:1000), anti-Bcl-2-associated X protein (Bax; cat. no. 505992-2-Ig; 1:2000), anti-adhesion-associated proteins intracellular adhesion molecule-1 (ICAM-1; cat. no. 10831-1-AP; 1:1000), anti-Wnt/β-catenin signal pathway-associated proteins (β-catenin cat. no. 51067-2-AP; 1:2000), anti-glucose-regulated protein 78 (GRP78; cat. no. 66574-1-Ig; 1:5000), and anti-GAPDH (cat. no. HRP-60004; 1:8000) antibodies were pauchased from ProteinTech Group, Inc. (chicago, IL, USA). Anti-phosphorylated β-catenin (cat. no. DF2989; 1:1,000) antibody was pauchased from Affnity Biosciences. (Cincinnati, OH, USA). Anti-vascular cell adhesion molecule-1 (VCAM-1; cat. no. WL02474; 1:500), anti-monocyte chemoattractant protein-1 (MCP-1; cat. no. WL01755; 1:1000) anti-activating transcription factor 6 (ATF6; cat. no. Wl02407; 1:800), and anti-C/EBP homologus protein (CHOP; cat. no. WL00880; 1:800) antibody was pauchased from Wanleibio. (Shenyang co., Ltd., China). Anti-low density lipoprotein receptor-related protein 6 (LRP6: cat. no. A13325; 1:1000) antibody was pauchased from ABclonal. (Wuhan co., Ltd., Wuhan, China). Anti-phosphorylated LRP6 (cat. no. abs140173) antibody was pauchased from Absin. (Shanghai co., Ltd., shanghai, China). Anti-mouse (cat. no. SA00001-1; 1:8000) secondary antibody was pauchased from ProteinTech Group, Inc. Horseradish peroxidase-conjugated anti-rabbit (cat. no. BA1054; 1:8000) was pauchased from Boster Biological Technology, Inc. (Wuhan, China).

### 4.7. LiCl or Salinomycin Treatment of HUVECs

HUVECs were plated in culture flasks. After reaching 70–80% confluency, the cells were treated with lithium chloride (LiCl, 40 µmol/L) or salinomycin (5 µmol/L) for 24 h and harvested. The following three experimental groups were established: C (untreated control cells), Sal (salinomycin-treated cells), and Li (LiCl-treated cells).

### 4.8. Sphingomyelin Synthase Enzyme Activity Assay

The HUVECs were treated with H_2_O_2_ for 24 h as previously described. The HUVECs were treated with 20 µmol/L Dy105 (provided by Dr. Deyong Ye, School of Pharmacy, Fudan University) for 24 h. The treated cells were incubated with NBD-ceramide (0.1 µg/µL, cat. no. 62527; Cayman chemical company, Ann Arbor, MI, USA) at 37 °C to synthesize sphingomyelin in vitro. After 3 h of incubation, the cells were harvested by 1520× *g* for 5 min, and the medium was collected at room temperature. According to the protein content in each group, the protein levels were adjusted to the volume of the reaction system (700 µL) to ensure consistency in the amount of total protein and medium added. The lipids were extracted in chloroform: Methanol (2:1) dried under N_2_ gas and separated by thin layer chromatography using chloroform:MeOH:NH4OH (14:6:1) at room temperature for 10 min [12]. The chromatography film was scanned after 10 min with an autoradiography system (Chemiluminescence Imaging System, Clinx Science Instruments Co., Ltd., Shanghai, China), and the intensity of each band was measured using Image-Pro Plus version 6.0 software (Media Cybernetics, Inc., Rockville, MD, USA) [12].

### 4.9. Statistical Analysis

The data were analyzed by GraphPad Prism 6.0 (San Diego, CA, USA). *t*-Tests were used for comparisons between two groups, and a one-way analysis of variance (ANOVA) was used for comparisons among multiple groups. All results were reduplicated at least thrice. *p* <0.05 was considered statistically significant. 

## 5. Conclusions

SMS2 1) triggers the Wnt/β-catenin pathway by increasing the phosphorylation of LRP6 and 2) promotes intracellular cholesterol accumulation, which contributes to the induction of ER stress and causes ED.
